# Development and validation of a highly effective analytical method for the evaluation of the exposure of migratory birds to antibiotics and their metabolites by faeces analysis

**DOI:** 10.1007/s00216-022-03953-4

**Published:** 2022-02-15

**Authors:** Carmen Mejías, Julia Martín, Juan Luis Santos, Irene Aparicio, Marta Isabel Sánchez, Esteban Alonso

**Affiliations:** 1 Departamento de Química Analítica, Escuela Politécnica Superior, Universidad de Sevilla, C/ Virgen de África, 7, 41011 Seville, Spain; 2 Departamento de Biología Vegetal Y Ecología, Facultad de Biología, Universidad de Sevilla, 41012 Seville, Spain; 3 Departamento de Ecología de Humedales, Estación Biológica de Doñana, CSIC. 41092 Seville, Spain

**Keywords:** Antibiotics, Metabolites, Wild bird faeces, LC–MS/MS, Analytical method

## Abstract

The widespread occurrence of antibiotics in the environment may exert a negative impact on wild organisms. In addition, they can become environmental reservoirs, through the ingestion of food or contaminated water, and vectors for antibiotic-resistant bacteria. This fact is even more important in migratory birds that can promote their dissemination across continents. In this work, a multiresidue analytical method suitable for the determination of five families of antibiotics and their main metabolites in waterbird faeces has been developed and validated. The target compounds include environmentally significant sulfonamides, macrolides, fluoroquinolones, tetracyclines and antifolates. Sample treatment involves ultrasound-assisted extraction with methanol and dispersive solid-phase extraction clean-up with C18. Analytical determination was carried out by liquid chromatography–tandem mass spectrometry. The most significant parameters affecting sample extraction and extract clean-up were optimised by means of experimental designs. Good linearity (*R*^2^ > 0.994), accuracy (from 41 to 127%), precision (relative standard deviation lower than 24%) and limits of quantification (lower than 2 ng g^−1^ (dry weight, dw)) were obtained for most of the compounds. The method was applied to the determination of the selected compounds in 27 faeces samples from three common migratory waterbird species. Nine antibiotics and three of their metabolites were detected in the analysed samples. Fluoroquinolones and macrolides were the antibiotics most frequently detected. The highest concentrations corresponded to norfloxacin (up to 199 ng g^−1^ dw).

**Graphical abstract:**

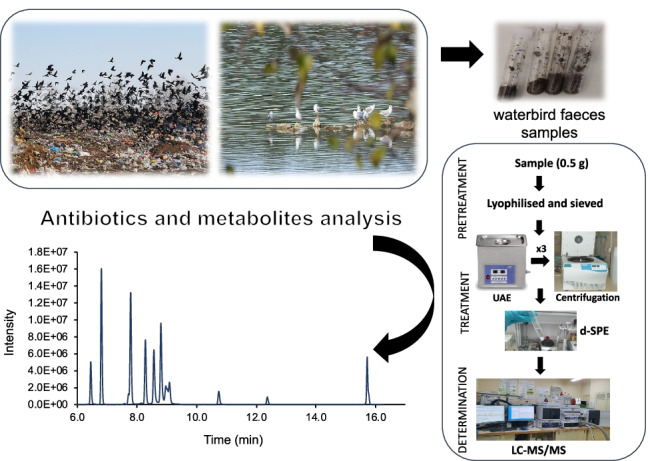

**Supplementary Information:**

The online version contains supplementary material available at 10.1007/s00216-022-03953-4.

## Introduction

The widespread use of antibiotics in human and veterinary medicine may lead to their environmental dissemination through discharges from wastewater treatment plants (WWTPs), animal farms and aquaculture ponds [1]. The presence of residues of fluoroquinolones, sulfonamides, tetracyclines or macrolides, among other antibiotics, has been reported in aquatic and terrestrial environmental compartments at part per billion or part per million levels [2–4]. In spite that their environmental concentrations are significantly lower than their therapeutic use concentrations, there is increasing evidence that such concentrations may cause antibiotic resistance which implies not only a threat to human and animal health but also a food safety risk [5–7]. This situation is even more worrying in the case of migratory birds because they can promote the international transfer of bacterial antibiotic resistance [8]. Migratory birds are considered a major source of the spread of antibiotic resistance across different environments such as water supplies and landfills as well as over long distances across habitats and regions [9–11]. Recently, Jarma et al. [9] found different bacterial communities in faeces from waterbirds wintering in Spain. Their results suggest that these birds may disseminate antibiotic resistance between landfills and natural habitats even in pristine environments such as Antarctica [12].

To assess the presence of antibiotics in birds, some methods have been proposed for their determination in feathers [13–20]. However, biomonitoring in feathers has several drawbacks such as being an invasive matrix that, foremost, requires bird capture which is costly and not always feasible. Moreover, such studies are limited to the determination of parent compounds not including the determination of metabolites. Metabolites of antibiotics can be present in wild birds not only because of metabolic transformations of the parent compounds but also by direct exposition via diet because of their presence in the aquatic and terrestrial environments. Therefore, analytical methods to evaluate the exposition of migratory birds to antibiotics should include not only parent compounds but also their metabolites. In addition, a non-invasive matrix such as faeces would be useful to evaluate risk assessment of wild birds, to calculate environmental loads and to evaluate environmental dissemination of antibiotic residues [9]. Analytical methods reported to date for the determination of pharmaceuticals in animal excrements have been focused on bovine manure [21–23], swine manure [21–28] and poultry excreta [2, 29, 30]. The aim of such studies is to evaluate the presence of veterinary antibiotics in manure or excreta not only because they can constitute a significant route of antibiotic dissemination into the environment but also because they could enter in the human food chain when manure is used in crops as low-cost organic fertilisers. Because of that, as can be seen above, most of the studies have been conducted in excrements from mammals, and much less from (domestic) birds. Nevertheless, analytical methods developed for their application to mammals’ excreta are not suitable for birds’ excreta because of the differences between both types of excreta. Whereas mammals excrete nitrogenous wastes mostly in the form of urea, birds convert it to uric acid or guanine, which is eliminated simultaneously with faeces through the cloaca (unlike mammals, which have separate mechanisms to eliminate faeces and urine) [31]. In addition, in spite that some analytical methods have been reported for the determination of pharmaceuticals in excreta from domestic birds, it can differ in their physical–chemical composition (based on the diet, flight activity, metabolism, physiological conditions, caecum microbiome, manipulated diet, etc.) from excreta from wild birds. For example, magnesium is commonly dietarily manipulated in domestic chickens to modify the consistency (moisture) of excreta [32]. Because of all the mentioned above, it is necessary to develop analytical methods for the specific determination of antibiotics and their metabolites in wild bird faeces as they constitute promising to non-invasive and easy to collect matrix to monitor exposure to antibiotics.

Therefore, the aim of this work was to optimise and validate an analytical method for the determination of twelve environmentally relevant antibiotics from five different families, and eight of their main metabolites, in wild bird faeces samples. Target compounds included three sulfonamides, four macrolides, three fluoroquinolones, a tetracycline, an antifolate and eight of their metabolites. The selection of the antibiotics was based on their presence in the environment [2–4]. Only one tetracycline was included in the analytical method because, in spite of being wide-spectrum antibiotics widely used in human and veterinary medicine, they are present at low levels in the aquatic environment as they precipitate with cations being retained onto wastewater treatment plants’ sewage sludge and sediments [2]. Sample treatment was based on easy-to-perform and low-cost techniques: ultrasonic-assisted extraction (UAE) and extract clean-up by dispersive solid-phase extraction (d-SPE). The method was applied to bird faeces from three different migratory waterbird species wintering in Doñana National Space and surrounding areas (Andalusia, South Spain). The application allowed us to evaluate the exposure of birds and their role as biovectors of antibiotic residuals, and to correlate these data with the diversity and abundance of the antibiotic resistance gene.

## Materials and methods

### Chemicals and reagents

Antibiotic standards of enrofloxacin (ENR, ≥ 98.5%), ciprofloxacin (CIP, ≥ 98.0%), sulfadiazine (SDZ, ≥ 99.0%), N^4^-acetylsulfadiazine (AcSDZ, > 99.0%), N^4^-acetylsulfamethoxazole (AcSMX, ≥ 98.5%), sulfamethazine (SMZ, ≥ 99.0%), roxithromycin (ROX, ≥ 98.0%) and tetracycline (TC, ≥ 95.0%) were purchased from Sigma-Aldrich (Steinheim, Germany). Sulfamethoxazole N^4^-glucoside (SMX-GL, > 99.0%), N^4^-acetylsulfamethazine (AcSMZ, ≥ 98.0%), N-desmethylclarithromycin (DM-CLM, ≥ 96.0%), 4-hydroxytrimethoprim (4-OH-TMP, ≥ 97.0%) and 3-desmethyltrimethoprim (DM-TMP, ≥ 98.0%) were supplied by Toronto Research Chemicals (Toronto, Canada). Clarithromycin (CLM, ≥ 98.0%) and erythromycin (ERY, ≥ 98.0%) were purchased from Tokyo Chemicals Industry (Eschborn, Germany). Trimethoprim (TMP, ≥ 99.5%), sulfamethoxazole (SMX, ≥ 99.5%) and norfloxacin (NOR, ≥ 99.1%) were supplied by Dr Ehrenstorfer GmbH (Augsburg, Germany). 4-Epitetracycline (EP-TC, > 99.0%) was provided from WHO Centre for Chemical Reference Substances (Strasbourg, France). Azithromycin (AZM, > 99.0%) was supplied by European Pharmacopoeia Reference Standard (Strasbourg, France). The internal standards (I.S.) ofloxacin-d_3_ (OFL-d_3_, ≥ 99.0%), demeclocycline (DMC, ≥ 90.0%), sulfamethoxazole-(phenyl-^13^C_6_) (SMX-^13^C, ≥ 99.0%) and erythromycin-(N,N-dimethyl-^13^C_2_) (ERY-^13^C, ≥ 99.0%) were purchased from Sigma-Aldrich (Steinheim, Germany). The structures of the target compounds, and their pK_a_ and log K_ow_ values, are presented in Table S1 in Electronic Supplementary Material (ESM).

Ammonium formate and Florisil® were supplied by Sigma-Aldrich (Madrid, Spain). Primary-secondary amine (PSA), ammonium acetate, C18 and glacial acetic acid were provided by Scharlab (Barcelona, Spain). Formic acid was provided by Panreac (Barcelona, Spain). The reagents were of high analytical grade and purity. LC–MS-grade acetonitrile (ACN), hexane, acetone, water and methanol (MeOH) were supplied by Biosolve BV (Valkenswaard, the Netherlands).

### Sample collection and treatment

Faeces samples corresponded to three migratory waterbird species: white stork (*Ciconia ciconia*), lesser black-backed gull (*Larus fuscus*) and black-headed gull (*Chroicocephalus ridibundus*). Freshly voided faeces were collected from roosting habitats in Doñana National Space (Andalusia, Spain). Monospecific flocks were previously located with binoculars. Then, we approached what caused birds to fly away, and then fresh faeces, easily distinguished from older ones, were collected from the core of the faecal material by using a spatula. To avoid collecting faeces from the same bird, samples were collected at least 2 m one from the other. They were transported to the laboratory in a cooler bag with a cooling block and then they were conserved at − 20 °C until analysis.

After collection, samples were freeze-dried in a Cryodos-50 lyophiliser (Telstar, Terrasa, Spain), homogenised in a mortar and sieved (particle size < 100 µm). Pre-treated faeces samples (0.5 g dry weight (dw)) were spiked with the I.S. (OFL-d_3_, DMC, SMX-^13^C and ERY-^13^C) at 100 ng g^−1^ dw. Samples were extracted three times with 5 mL of MeOH by sonication in an ultrasonic bath (25 °C, 80 kHz) for 10 min. After each extraction, the solid–liquid separation was carried out by centrifugation for 10 min at 2900 × *g*. The liquid phases were combined into a clean centrifuge tube containing 0.8 g of C18 for d-SPE clean-up. The tubes were shaken and centrifuged for 10 min at 2900 × *g*. The liquid phase was transferred to another tube to be evaporated to dryness under a gentle nitrogen stream. The extract was reconstituted in 0.5 mL of MeOH:water (1:1, v/v) and filtered through a 0.22 µm cellulose syringe filter. A 2 µL aliquot of the filtered extract was injected into the liquid chromatography–tandem mass spectrometry (LC–MS/MS) instrument.

### Instrumental analysis

Chromatographic determination was performed using an Agilent 1260 Infinity II chromatograph (Agilent, Santa Clara, CA, USA). Chromatographic separation was carried out in a Zorbax RRHD Eclipse Plus C18 (150 mm × 3.0 mm i.d., 1.8 μm particle size) column (Agilent, Santa Clara, CA, USA), protected with a Zorbax RRHD Eclipse Plus C18 (3.0 mm i.d., 1.8 µm particle size) guard column (Agilent, Santa Clara, CA, USA). The mobile phase was composed of a 10 mM ammonium formate buffer containing 0.05% of formic acid (solvent A) and MeOH (solvent B). Elution was carried out at a flow rate of 0.4 mL min^−1^ with chromatographic column thermostated at 35 °C. Elution started with 5% of solvent B, held 1 min. Solvent B was linearly increased to 30% in 3 min, then to 60% in 8 min and, finally, to 100% in 2 min, held for 2 min. Back to initial conditions was carried out in 2 min and held for 2 min for equilibration. The total run time was 20 min.

The LC system was coupled to a 6495 triple quadrupole mass spectrometer (MS/MS) equipped with an electrospray ionisation source operated in positive mode. The following settings were used: fragmentor, 166 V; capillary voltage, 4000 V; nebuliser pressure, 40 psi; sheath gas flow rate, 12 L min^−1^, sheath gas temperature, 250 °C; drying gas flow rate, 11 L min^−1^ and gas temperature, 350 °C.

## Results and discussion

### LC–MS/MS optimisation

LC–MS/MS parameters were optimised by injection of individual and mixture standard solutions of the selected compounds at 1 mg L^−1^. The type and composition of mobile phase solvents were optimised to achieve the highest compound ionisation to improve analytical signals and lower limits of detection. First, the aqueous phase (solvent A) was optimised using MeOH as organic solvent (solvent B). Ammonium formate and ammonium acetate, at different concentrations (from 2 to 10 mM) and with different additives (formic acid for ammonium formate and acetic acid for ammonium acetate (from 0 to 0.2% v/v)), were tested. The optimisation was carried out in both positive and negative modes. For all the compounds, the best results were obtained in positive mode. The precursor ions corresponded to the molecular ions after protonation. The highest intensities were provided by ammonium formate 10 mM containing 0.05% v/v of formic acid so that mixture was selected as aqueous mobile phase solvent. In all cases, the two most abundant product ions were monitored for each compound, one for quantification and the other for confirmation.

Then, the use of ACN as an organic solvent instead of MeOH was tested. As no improvement was observed, MeOH was selected due to its lower price and toxicity in comparison to ACN [33]. The flow rate was optimised in the range from 0.3 to 0.6 mL min^−1^ to reduce run time as much as possible at acceptable peak resolution. A flow rate of 0.4 mL min^−1^ was selected because it provided good resolution with low column pressure.

The optimised LC–MS/MS parameters for each compound are given in Table 1. The analyses were carried out using dynamic multiple reaction-monitoring mode (dMRM).

**Table 1:** LC–MS/MS conditions and retention times for the target compounds

Group	Compound	Internal standard	Precursor ion (*m/z*)	Product ions (quantifier/qualifier) (*m/z*)	CE (eV)	Ratio	Retention time (min)
Macrolides	RXM	ERY-^13^C	838.1	158.1/679.4	32/20	72.9	15.76
	AZM	ERY-^13^C	750.0	591.5/116.1	28/44	56.1	12.35
	ERY	ERY-^13^C	734.5	83.0/576.4	68/20	81.4	15.71
	CLM	ERY-^13^C	749.0	158.1/590.4	28/16	49.5	15.72
	DM-CLM	ERY-^13^C	734.9	144.1/576.4	24/16	23.5	15.72
Fluoroquinolones	NOR	OFL-d_3_	320.3	302.1/231	24/44	26.0	8.56
	ENR	OFL-d_3_	360.4	286.1/342.1	40/40	61.7	9.08
	CIP	OFL-d_3_	332.1	314.1/231	16/40	98.5	8.79
Tetracyclines	TC	DMC	445.4	410.2/154.1	20/28	57.9	9.05
	EP-TC	DMC	445.4	410.2/98.1	20/48	27.0	8.13
Antifolates	TMP	SMX-^13^C	291.2	261.1/229.8	28/24	98.2	7.79
	4-OH-TMP	SMX-^13^C	279.2	93.0/121.1	40/40	1.10	8.27
	DM-TMP	SMX-^13^C	277.3	261.4/123.0	28/44	63.1	6.80
Sulfonamides	SMX	SMX-^13^C	254.3	92.1/108.0	28/28	76.1	8.96
	AcSMX	SMX-^13^C	296.3	134.0/108.1	24/28	49.8	10.74
	SMX-GL	SMX-^13^C	416.4	254.0/108.0	8/44	9.50	7.59
	SDZ	SMX-^13^C	251.3	92.1/156.0	28/12	98.0	6.45
	AcSDZ	SMX-^13^C	293.3	134.1/198.0	24/16	74.9	7.71
	SMZ	SMX-^13^C	279.3	186.0/92.0	16/36	76.4	8.28
	AcSMZ	SMX-^13^C	321.4	186.0/134.0	20/28	81.3	9.08
Internal standards	DMC	-	465.1	448.1/430.1	16/24	61.3	10.18
	ERY-^13^C	-	736.9	160.1/578.4	32/16	56.2	15.31
	OFL-d_3_	-	365.4	321.2/261.1	20/28	90.7	8.25
	SMX-^13^C	-	260.2	98.1/162.0	32/16	94.5	8.95

*CE* collision energy

### Method optimisation

The most significant parameters affecting UAE (type, acidification and volume of the extraction solvent, extraction time and number of extraction cycles) and d-SPE clean-up (type and amount of sorbent) were evaluated. Optimisation was carried out with faeces (0.5 g dw) spiked to a final concentration of 100 ng g^−1^ dw for each of the target compounds. The spiked faeces were incubated in the dark for 12 h to allow equilibration. Experiments were carried out in triplicate.

#### Extraction solvent optimisation

Four solvents (ACN, acetone, MeOH and hexane) were tested. Samples were ultrasonicated for 10 min using 3 mL of the tested solvent. Then, extracts were subjected to d-SPE clean-up by the addition of C18 (0.8 g). To calculate extraction recoveries, signals obtained from spiked faeces samples were compared with those from a matrix-matched standard at the same concentration.

**Fig. 1:**
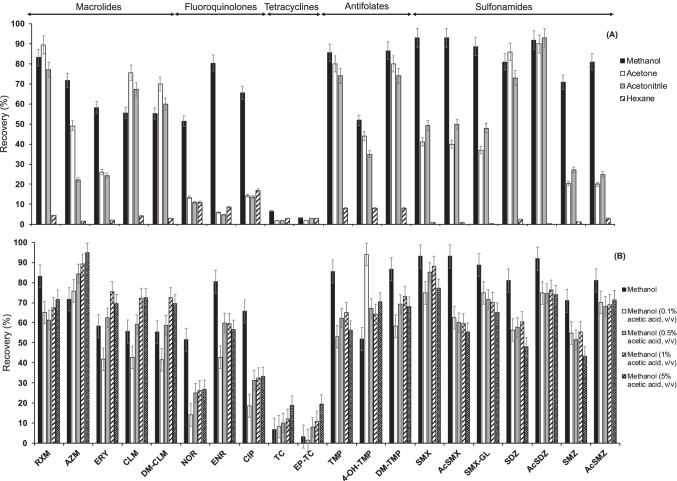
Optimisation of **A** extraction solvent and **B** acidity of the extraction solvent. Bars indicate standard deviation errors (*n* = 3)

As can be seen in Fig. 1, the best results were achieved with MeOH (mean recoveries were 75% except for tetracyclines), followed by acetone (49%), ACN (46%) and hexane (5%). The chemical structures of antibiotics are generally more complex and bigger than those of other pharmaceuticals. They have several ionisable functional groups and variable water solubilities and polarities (Table S1 in ESM). The low recoveries achieved for tetracyclines, with all the tested solvents, could be explained by the formation of complexes with bi- and trivalent cations present in the matrix, as was described previously [23, 34].

The second optimisation experiment comprised the study of the influence of the acidification of MeOH (up to 5% v/v of acetic acid). Acidified MeOH increased the signals from tetracyclines and their metabolite which can be explained by their acidic properties. However, the addition of acetic acid has a negative effect on neutral and basic antibiotics, mainly for AcSMX that suffer a recovery decrease from 30 to 35%. Sulfonamides, fluoroquinolones and antifolates (except 4-OH-TMP) presented the best extraction recoveries when pure MeOH was used whereas macrolides and their metabolites had not a common optimal extraction solvent. In addition, it has been reported that acid media, and the presence of buffers like citrate, can produce the epimerisation of tetracyclines [23], TC to EP-TC, resulting in alterations in the concentrations of each compound. Tetracyclines were poorly extracted. Nevertheless, no other additives were tested as pure MeOH provided good extraction recoveries for most of the antibiotics. It was selected as an extraction solvent.

#### Clean-up optimisation: type and amount of d-SPE sorbent

**Fig. 2:**
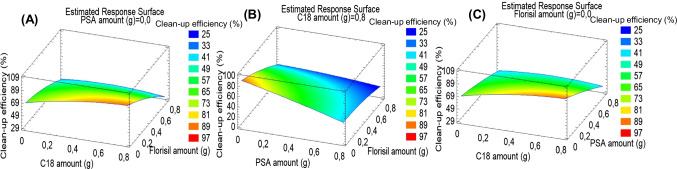
Response surface plots corresponding to mean clean-up efficiency when optimising the following pair of d-SPE factors: **A** C18 vs Florisil®; **B** PSA vs Florisil®; **C** C18 vs PSA. Faeces samples were spiked at 100 ng g^−1^ dw for each pollutant

Clean-up was optimised in order to choose the most suitable sorbent, or sorbents, to remove interfering compounds without removing target compounds. Three sorbents (C18, PSA and Florisil®) and three sorbent amounts (0, 0.4 and 0.8 g) were simultaneously evaluated using a Box-Behnken experimental design (BBD). Samples were extracted by sonication for 10 min using 3 mL of MeOH. Extracts were subjected to the 15 clean-up experiments generated by the BBD matrix (see Table S2 in ESM). Clean-up efficiency was evaluated by comparing signals obtained after clean-up of spiked matrix extracts with those from a standard solution in MeOH:water (1:1, v/v) at the same concentration. Similar response surface plots were obtained from the five antibiotic families. In Fig. 2, it can be seen response surface plots corresponded to the average clean-up efficiency (%). Poor clean-up efficiency was obtained with PSA and Florisil®. The best results were obtained with 0.8 g of C18. Therefore, the conditions were selected for d-SPE extract clean-up.

#### UAE optimisation: solvent volume, extraction time and number of extraction cycles

**Fig. 3:**
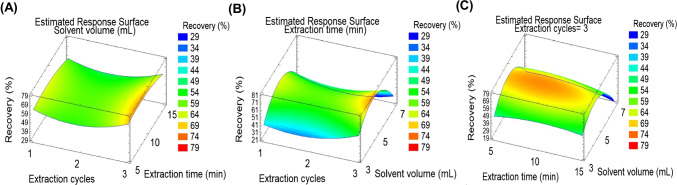
Response surface plots corresponding to mean method recovery when optimising the following pair of UAE factors: **A** number of extraction cycles vs extraction time; **B** number of extraction cycles vs MeOH volume; **C** extraction time vs MeOH volume. Faeces samples were spiked at 100 ng g^−1^ dw for each pollutant

Extraction time, solvent volume and number of extraction cycles were optimised by BBD. Three levels were evaluated for each variable: extraction time (5, 10 and 15 min), extraction solvent volume (3, 5 and 7 mL) and number of extraction cycles (1, 2 and 3). A total of 15 extraction experiments were performed (see Table S3 in ESM). After each extraction experiment, extract clean-up with 0.8 g of C18 was applied. Response surface plots, corresponding to mean method recovery (%), were constructed to better evaluate the effects of each variable and their interactions (Fig. 3). The most significant parameter was the number of extraction cycles. Three extraction cycles were necessary to quantitatively extract antibiotics and their metabolites. The best results for extraction time and MeOH volume were 10 min and 5 mL, respectively.

### Method validation

The method was validated in terms of matrix effect (ME), linearity, sensitivity, accuracy, precision and selectivity. ME was assessed by comparison of the calibration curve slopes in matrix-matched standards and in pure standard solutions in MeOH:water (1:1, v/v). Eight-point calibration curves were prepared in the range from method quantification limits (MQL) to 200 ng g^−1^ dw. Student’s *t*-test, at 95% of confidence, revealed significant differences between curve slopes which confirmed the presence of ME. Therefore, matrix-matched calibration was applied for the quantification of all the analytes. Their determination coefficients (*R*^2^) were higher than ≥ 0.994 for all the compounds (Table 2). ME was quantified by comparison of the peak area of the target compounds in matrix extract (*A*_extract_), after subtracting the peak area obtained from non-spiked extracts (*A*_blank_), and in pure solvent standard solutions (*A*_standard_) following equation: ME (%) = (*A*_*extract*_—*A*_*blank*_—*A*_*standard*_)/*A*_*standard*_ × 100). ME was quantified at three concentration levels: 5, 50, and 100 ng g^−1^ dw, except for 4-OH-TMP, TC and EP-TC which concentration levels were 75, 100 and 200 ng g^−1^ dw, respectively. Both matrix suppression and enhancement were obtained in faeces samples, especially for tetracycline family. RMX, CLM, DM-CLM, TMP, 4-OH-TMP, DM-TMP, SMX, SDZ, ERY and SMZ suffered matrix suppression while AZM, TC, EP-TC, AcSMX, SMX-GL, AcSDZ, NOR, ENR, CIP and AcSMZ suffered matrix enhancement effect in bird faeces matrix.

**Table 2:** Recovery (*R*), accuracy (*A*), matrix effect (ME), precision (RSD), method detection (MDL) and quantification (MQL) limits, and matrix-matched calibration curve correlation coefficients (*R*^2^)

Compound	Low level				Medium level				High level				MDL (ng g^−1^ dw)	MQL (ng g^−1^ dw)	*R*^2^
	*R* (%)	*A* (%)	ME (%)	RSD (%)	*R* (%)	*A* (%)	ME (%)	RSD (%)	*R* (%)	*A* (%)	ME (%)	RSD (%)			
RXM	58.6	96.4	− 69.7	1.9	69.3	84.7	− 66.1	20.3	36.8	98.6	− 55.5	10.9	0.04	0.08	0.995
AZM	96.4	89.8	17.7	0.9	100.5	68.4	2.3	9.7	48.7	104.2	46.0	13.3	0.01	0.03	0.994
ERY	82.3	45.9	− 82.4	1.7	112.5	69.7	− 59.5	1.9	97.3	88.0	− 49.0	5.5	0.61	1.22	0.996
CLM	97.3	80.8	− 80,8	0.5	108.6	42.4	− 72.4	8.8	53.8	88.2	− 52.0	7.6	0.03	0.05	0.998
DM-CLM	85.1	47.0	− 85.6	2.0	107.3	41.2	− 73.3	10.2	58.9	83.6	− 58.9	5.8	0.03	0.06	0.996
NOR	77.6	86.4	4.2	9.4	46.8	87.2	22.2	11.8	99.2	54.1	53.0	5.2	0.03	0.06	0.999
ENR	73.3	106.4	103.1	3.9	84.1	118.8	53.9	17.3	86.9	112.6	55.2	9.4	0.07	0.14	0.997
CIP	63.9	74.6	0.2	11.9	30.7	104.9	3.4	17.3	79.1	50.9	68.2	5.5	0.04	0.08	0.999
TC*	27.3	123.7	31.6	2.8	29.1	91.9	162.8	2.1	30.9	60.1	131.2	1.3	1.00	25.0	0.997
EP-TC*	40.1	110.6	17.7	4.9	38.3	126.5	113.9	4.8	36.5	94.7	210.1	4.7	15.00	50.0	0.995
TMP	68.1	80.3	− 73.4	1.9	80.2	88.8	− 61.9	9.6	77.5	93.2	− 55.3	8.3	0.07	0.15	0.994
4-OH-TMP*	54.0	81.4	− 41.9	9.5	56.8	84.0	− 38.5	10.4	59.5	86.5	− 35.0	11.2	15.0	50.0	0.996
DM-TMP	94.8	95.6	− 73.3	2.9	101.7	56.9	− 54.1	13.9	68.9	82.0	− 31.3	7.9	0.05	0.10	0.994
SMX	52.6	104.9	− 35.0	24.0	29.7	92.9	− 42.0	15.6	68.0	80.8	− 69.3	12.4	1.43	1.81	0.998
AcSMX	13.3	56.0	13.9	0.4	76.8	102.5	3.6	9.1	76.6	95.6	1.7	5.5	0.19	0.38	0.998
SMX-GL	35.2	105.6	83.6	23.5	74.1	106.3	36.2	9.6	69.4	98.4	27.1	2.1	0.50	1.00	0.994
SDZ	35.6	64.3	− 61.1	6.8	73.4	70.6	− 47.9	4.5	59.2	88.8	− 41.7	10	0.70	1.40	0.999
AcSDZ	45.8	90.4	62.3	13.6	85.9	95.4	26.4	8.2	74.1	98.7	38.6	7.9	0.56	1.00	0.995
SMZ	50.9	77.3	− 38.4	1.6	57.5	102.1	− 30.4	7.6	58.2	98.4	− 29.6	10.9	1.47	1.96	0.995
AcSMZ	42.5	93.8	31.1	9.7	79.2	100.5	37.9	8.9	76.0	97.5	36.6	5.8	1.18	1.76	0.995

Spiking levels: 5, 50 and 100 ng g^−1^ dw for low, medium and high level, respectively; *spiking levels: 75, 100 and 200 ng g^−1^ dw for low, medium and high level, respectively

Sensitivity was assessed in terms of method detection limits (MDL) and MQL. These parameters were calculated as the concentrations provided signal to noise ratios of 3 and 10 for MDL and MQL, respectively. MQL values were lower than 2 ng g^−1^ dw except for TC (25 ng g^−1^ dw), 4-OH-TMP (50 ng g^−1^ dw) and EP-TC (50 ng g^−1^ dw). Table 2 shows obtained values. The extraction recoveries, accuracies, and precision of the method were evaluated using spiked faeces samples at three concentration levels in triplicate. Spike concentrations were 5 ng g^−1^ dw (low), 50 ng g^−1^ dw (medium) and 100 ng g^−1^ dw (high), for compounds with MQL values higher than 2 ng g^−1^ dw; and 75 ng g^−1^ dw (low), 100 ng g^−1^ dw (medium) and 200 ng g^−1^ dw (high) for TC, EP-TC and 4-OH-TMP. Extraction recoveries (*R*) were calculated by comparison of the peak areas obtained from the spiked samples (*A*_sample_) with those from spiked extracts (*A*_extract_), after blank correction (*A*_blank_) applying equation: *R* (%) = (*A*_*sample*_—*A*_*blank*_)/(*A*_*extract*_—*A*_*blank*_) × 100. Accuracy (*A*), expressed as relative recovery, was determined by comparison of the concentration obtained from spiked samples using matrix-matched calibration curves (*C*_spiked sample_), after blank correction (*C*_blank_), with the spike concentration (*C*_spike concentration_) applying equation: *A* (%) = (*C*_*spiked sample*_—*C*_*blank*_) × 100/R (%) × 100/*C*_*spike concentration*_. Results are shown in Table 2. Accuracies at the three spike concentrations were in the range from 42.4 to 104.2% for macrolides, 50.9–118.8% for fluoroquinolones, 60.1–126.5% for tetracyclines, 56.9–101.7% for antifolates and 56.0–106.3% for sulfonamides. Precision was calculated as inter-day repeatability and expressed as relative standard deviation (RSD, %). RSD values were below 24% for all compounds at the three spike concentrations. Method selectivity was evaluated by comparison of the chromatograms of procedural blanks and spiked faeces samples. No interference was observed at the retention times of the target compounds. In Fig. S1 (ESM), it can be seen a LC–MS/MS chromatogram of a faeces sample spiked at 75 ng g^−1^ dw.

### Method comparison

In Table 3, it is summarised a comparison between the operational and analytical parameters of the proposed method and those reported in the literature for the determination of the target compounds in excreta. As mentioned in “Introduction,” such methods were developed for the determination of veterinary antibiotics in excreta and manure from farm animals, mainly mammals, not for environmentally relevant antibiotics in wildlife birds. Wild animals, and particularly synanthropic birds, are overlooked but key agents in the epidemiology of clinically important antibiotic-resistant bacteria [35, 36], so a specific analytical method for the determination of environmentally relevant antibiotics in wild bird faeces is highly required. The proposed extraction methods are mainly based on UAE [25, 28–30, 37] and on solid–liquid extraction (SLE) [22–24, 26, 38] followed by extract clean-up by SPE [21, 22, 26, 27, 29, 37]. QuEChERS (quick, easy, cheap, effective, rugged and safe) method [39] and pressurised liquid extraction (PLE) [21] have been also proposed. The proposed method allows several operational advantages such as a low sample mass (0.5 g), which is mandatory for the analysis of bird faeces; low-cost instrumentation in comparison to the PLE method; lower solvent consumption (15 mL) in comparison to QuECheRS, SPE clean-up and some of the proposed UAE and SLE methods (up to 144 and 317 mL, respectively); and no plastic waste generation in comparison to clean-up by SPE cartridges or QuECheRS method. Accuracy values are similar or even better than the above-mentioned methods. MDLs values achieved are lower (from 0.01 to 1.5 ng g^−1^ dw for 90% of the target compounds) than those reported by other authors (Table 3) especially in comparison to methods developed for antibiotics from different families for which MDLs up to 500 ng g^−1^ dw have been reported [39]. For instance, the method proposed by Pokrant et al. [40], for the determination of veterinary antibiotics in broiler chicken faeces, achieved MDLs in the range from 17.5 to 37. 4 ng g^−1^ in spite of a higher sample amount being treated (1 g) in comparison to the proposed method (0.5 g). Such MDLs were suitable for the determination of antibiotics after administration to chickens as their concentrations in faeces were in the range of 68 to 2058 ng g^−1^. Nevertheless, lower MDLs are required for the determination of environmentally relevant antibiotics in wild bird faeces. Furthermore, the proposed method allows the determination of 8 metabolites whereas only four of the published methods include the determination of metabolites of antibiotics but just two or three metabolites and from one or two families of antibiotics (sulfonamides [23]; tetracyclines [23, 27, 40]; or fluoroquinolones [37]). These facts are of special relevance as concentrations of pharmaceuticals in wild bird faeces are expected to be lower than in livestock and poultry excrements as antibiotics are intendedly administered to farm animals; and because bird animals can be directly exposed not only to parent compounds but also to metabolites present in the environment.

**Table 3:** Summary of analytical methods published in the last 10 years for the determination of antibiotics and their metabolites in animals excrements

Antibiotics and metabolites	Matrix	Sample amount (g)	Extraction technique	Extraction solvent volume (mL)	Clean-up	Analytical determination	Accuracy (%)	MDL (ng g^−1^ dw)	Reference
2 macrolides, 1 antifolate, 1 sulfonamide, 2 fluoroquinolones and 1 tetracycline	Broiler manure	1	UAE	26	SPE	LC–MS/MS	63–113	1–5	[29]
10 sulfonamides, 4 tetracyclines and 2 *metabolites*	Cattle faeces and swine liquid manure	1	SLE	6.2	-	LC–MS/MS	70–130	10–80	[23]
2 fluoroquinolones and 2 *metabolites*	Chicken manure	20	UAE	144	SPE	UHPLC-MS/MS	-	-	[37]
3 sulfonamides and 1 tetracycline	Cow excrements	0.5	SLE	17.4	-	LC–MS/MS	70–130	10–80	[38]
1 tetracycline	Liquid pig manure	25	SLE	100	LLE + SPE	LC–MS/MS	51–87	-	[26]
8 macrolides, 5 nitroimidazoles, 3 amphenicols and 17 sulfonamides	Livestock and poultry excrement	0.5	PLE	-	d-SPE	UHPLC-MS/MS	60.7–103.9	0.4–3.5	[21]
4 sulfonamides	Pig excreta	10	SLE	40.2	-	LC-FLD	78–99	0.6–2.8	[24]
5 sulfonamides, 4 fluoroquinolones, 3 tetracyclines, 2 β-lactams, 2 macrolides, 1 pleuromutilins and 1 amphenicol	Pig manure	0.3	In situ d-SPE and UAE	31	c-SPE	UHPLC-MS/MS	92–106	0.02–136 (MQL)	[25]
8 β-Lactams, 24 sulfonamides, 23 fluoroquinolones, 20 imidazoles, benzimidazoles, 14 benzimidazoles, 5 polyethers and 12 macrolides	Pig, cattle and chicken faeces	2	QuEChERS method	22	SPE and EMR-Lipid	LC-QTOF-MS	75–99	0.8–500	[39]
2 tetracyclines, 2 β-lactams, 3 fluoroquinolones, 3 sulfonamides, 1 antifolate and 1 polymyxins	Poultry excreta	1	UAE	10	-	UHPLC-MS/MS	89.2–107.8	2.19–9.22	[30]
4 tetracyclines, 18 sulfonamides, 14 macrolides and 10 quinolones	Swine and calf faeces	2	SLE	12	SPE	LC–MS/MS	83.7–147	0.5–32	[22]
3 tetracyclines and 3 sulfonamides	Swine faeces	0.2–0.5	UAE	70	SPE	UHPLC-MS/MS	69.1–140	0.35–18	[28]
4 tetracyclines and 2 *metabolites*	Swine manure	1	SLE	10	SPE	UHPLC-MS/MS	64–112	1.9–7.3 ng mL^−1^	[27]
3 tetracyclines, 1 macrolide, 3 fluoroquinolones, 1 phenicols, 2 sulfonamides and 3 *metabolites*	Chicken faeces	1	SLE	10	SPE	LC–MS/MS	91.9–104.1	17.5–37.4	[40]
3 sulfonamides, 4 macrolides, 3 fluoroquinolones, 1 tetracycline, 1 antifolate and 8 *metabolites*	Bird faeces	0.5	UAE	15	d-SPE	LC–MS/MS	41–127	0.01–15	Proposed method

*c-SPE* compact solid-phase extraction; *EMR-Lipid* enhanced matrix removal-lipid; *LC-FLD* liquid chromatography with fluorescence detection; *LC–MS/MS* liquid chromatography–tandem mass spectrometry; *LC-QTOF-MS* liquid chromatography–quadrupole time-of-flight mass spectrometry; *LLE* liquid–liquid extraction; *QuEChERS* quick, easy, cheap, effective, rugged & safe extraction; *SPE* solid-phase extraction; *SLE* solid–liquid extraction; *PLE* pressurized-liquid extraction; *d-SPE* dispersive solid-phase extraction; *UAE* ultrasound-assisted extraction; *UHPLC-MS/MS* ultra-high-performance liquid chromatography–tandem mass spectrometry

### Method application

The developed method was applied to the determination of the target compounds in 27 faeces samples from three waterbirds species wintering in Spain: *Ciconia ciconia* (*n* = 15), *Larus fuscus* (*n* = 8) and *Chroicocephalus ridibundus* (*n* = 4). The results obtained are shown in Table S4 (ESM). A summary of the obtained results can be seen in Table 4. Nine parent compounds and three metabolites were found in analysed samples. Parent compounds were more frequently detected and at similar or higher concentrations than their metabolites, except for TC. The metabolite of TC (EP-TC)) was detected in just four faeces’ samples, all of them from the white stork *Ciconia ciconia* whereas TC was detected in no sample. It has been described that tetracyclines are instable compounds that can suffer abiotic degradation in the environment, conditioned by temperature, pH, light and redox conditions, being EP-TC one of the main degradation products [41]. Therefore, the detection of EP-TC in four faeces samples, whereas TC was not detected, could be due to a high TC metabolisation in white stork *Ciconia ciconia* or to a higher ingestion of EP-TC released to the environment through wastewater or generated as a degradation product in the environment and landfills. Nevertheless, more samples should be analysed to obtain concluding results. RXM, NOR and TMP were detected in all the analysed samples and bird species. AZM was detected in all samples, except *Chroicocephalus ridibundus* samples. Fluoroquinolones and macrolides were the families most frequently detected. The highest concentrations belonged to NOR (up to 199.27 ng g^−1^ in *Ciconia ciconia* faeces sample 9), SMX (up to 300.62 ng g^−1^ dw in *Ciconia ciconia* faeces sample 3) and AcSMX (up to 148.02 ng g^−1^ dw in *Larus fuscus* faeces sample 8). ERY, TC, 4-OH-TMP, SMX-GL, SMZ, AcSMZ and AcSDZ were not detected in any of the analysed samples.

**Table 4:** Range, mean concentration and frequency of detection (Freq.) of antibiotics and metabolites measured in bird faeces samples from Doñana National Park

Compound	*Ciconia ciconia* (*n* = 15)			*Larus fuscus* (*n* = 8)			*Chroicocephalus ridibundus* (*n* = 4)		
	Range (ng g^−1^ dw)	Mean (ng g^−1^ dw)	Freq (%)	Range (ng g^−1^ dw)	Mean (ng g^−1^ dw)	Freq (%)	Range (ng g^−1^ dw)	Mean (ng g^−1^ dw)	Freq (%)
RXM	0.21–1.91	0.64	100	0.37–0.80	0.58	100	0.2–0.36	0.26	100
AZM	0.66–2.44	0.99	100	0.69–1.51	0.84	100	< MDL	< MDL	0
ERY	< MDL	< MDL	0	< MDL	< MDL	0	< MDL	< MDL	0
CLM	0.05–0.42	0.17	27	0.06–0.08	0.07	50	< MDL	< MDL	0
DM-CLM	0.08–0.34	0.21	13	0.09	0.09	12	< MDL	< MDL	0
NOR	7.43–199	26.9	100	8.21–17.5	11.1	100	3.87–7.43	5.00	100
ENR	2.92–22.9	6.49	93	2.51–5.18	3.32	100	6.31–27.2	13.0	100
CIP	14.3–47.0	28.4	93	8.24–15.7	11.3	100	< MDL	< MDL	0
TC	< MDL	< MDL	0	< MDL	< MDL	0	< MDL	< MDL	0
EP-TC	56.0–59.2	57.9	27	< MDL	< MDL	0	< MDL	< MDL	0
TMP	0.37–1.82	1.03	100	0.7–3.72	1.50	100	0.19–0.40	0.30	100
4-OH-TMP	< MDL	< MDL	0	< MDL	< MDL	0	< MDL	< MDL	0
DM-TMP	< MDL	< MDL	0	< MDL	< MDL	0	< MDL	< MDL	0
SMX	2.14–300	54.5	53	2.19–14.3	6.12	88	< MDL	< MDL	0
AcSMX	2.54–2.57	2.56	13	27.0–148	87.5	25	< MDL	< MDL	0
SMX-GL	< MDL	< MDL	0	< MDL	< MDL	0	< MDL	< MDL	0
SDZ	6.53	6.53	7	4.81	4.81	12	4.26	4.26	25
AcSDZ	< MDL	< MDL	0	< MDL	< MDL	0	< MDL	< MDL	0
SMZ	< MDL	< MDL	0	< MDL	< MDL	0	< MDL	< MDL	0
AcSMZ	< MDL	< MDL	0	< MDL	< MDL	0	< MDL	< MDL	0

< *MDL* lower than the limit of detection of the method

Concentration levels measured are consistent with data available in faeces and manure samples from farm animals. For instance, Wang et al. [21] reported concentrations of RXM and SDZ up to 5.1 and 23.7 ng g^−1^ dw, respectively, in livestock and poultry excrements. Berendsen et al. [22] reported concentrations of CIP up to 13 ng g^−1^ dw in cattle faeces and Argüeso-Mata et al. [25] found concentrations of SMX up to 70 ng g^−1^ dw in pig manure. The high concentrations of antibiotics in the studied birds can be explained by their omnivorous and opportunistic habits. White storks (*Ciconia ciconia*), lesser black-backed gulls (*Larus fuscus*) and black-headed gulls (*Chroicocephalus ridibundus*) commonly use landfills and wastewaters for feeding, which have been reported to constitute an important reservoir of antibiotics [42]. This fact is consistent with results from a previous work in which it has been observed that faeces from storks and gulls contained a significantly higher abundance of antibiotic resistance genes than faeces from geese and cranes that feed in more natural habitats [9]. Moreover, the studied birds can acquire antibiotics by ingestion of preys where antibiotics can be bioaccumulated as reported for many wild-living aquatic organisms [43]. In addition, a concentration effect can occur in faeces, with respect to the amounts of the antibiotics in water and food ingested by the migratory birds.

## Conclusions

An analytical method for the determination of five antibiotic families and their main metabolites in bird faeces samples has been optimised and validated. To the date and to the best of our knowledge, the proposed method constitutes the first one for multiresidue determination of different families of antibiotics and their main metabolites in wild bird faeces samples. This fact is of especial relevance not only because the composition of wild bird faeces is different from poultry excrements but also because antibiotics to which wild birds are exposed can be different to antibiotics administered to poultry.

In addition, this is the first method combining UAE and d-SPE for the determination of antibiotics in faeces samples. The method allowed good linearity (*R*^2^ ≥ 0.994), accuracy close to 100%, adequate precision (RSD < 24%) and low MQLs (< 2 ng g^−1^ dw) for most of the compounds. Recoveries and MDLs were similar or improved than those reported for other excrement matrices but requires lower solvent volumes and sample amounts.

The analysis of 27 faeces samples from three common migratory waterbirds species revealed the presence of 9 out of the 12 antibiotics and 3 of their main metabolites. The proposed method can be a useful tool not only to monitor environmental risks for wild waterbirds but also (i) to assess biovectoring and quantification of antibiotic load into specific environments; (ii) to evaluate the environmental risk caused by the environmental dissemination of residues of antibiotics; and (iii) as a “tool” to reveal the overuse of antibiotics and their environmental load. This information is increasingly important due to the need for a better integration of wildlife into the current One Health approach for antibiotic resistance surveillance and control.

## Supplementary Information

Below is the link to the electronic supplementary material.

Supplementary file1 (DOCX 411 KB)
