# Supplementary material for: Development and validation of a highly effective analytical method for the evaluation of the exposure of migratory birds to antibiotics and their metabolites by faeces analysis
Source: Anal Bioanal Chem. 2022 Feb 15;414(11):3373–86. doi: 10.1007/s00216-022-03953-4 (PMC9018661; doi:10.1007/s00216-022-03953-4)
Supplement: Supplementary file 1 — Supplementary file1 (DOCX 411 KB) [file 216_2022_3953_MOESM1_ESM.docx]

**Development and validation of a highly effective analytical method for the evaluation of the exposure of migratory birds to antibiotics and their metabolites by faeces analysis**

Carmen Mejías^1^, Julia Martín^1^, Juan Luis Santos^1^*, Irene Aparicio^1^, Marta Isabel Sánchez^2,3^, Esteban Alonso^1^

^1^Departamento de Química Analítica, Escuela Politécnica Superior, Universidad de Sevilla. E-41011 Seville, Spain.

^2^Departamento de Biología Vegetal y Ecología, Facultad de Biología, Universidad de Sevilla. E-41012 Seville, Spain.

^3^Departamento de Ecología de Humedales, Estación Biológica de Doñana, CSIC. E-41092 Seville, Spain.

Analytical and Bioanalytical Chemistry

*Corresponding author

*Address*: Juan Luis Santos Morcillo

Departamento de Química Analítica

Escuela Politécnica Superior

Universidad de Sevilla

c/ Virgen de África, 7

41011 Seville (Spain)

*E-mail address*: jlsantos@us.es

**Table S1.** Physical-chemical properties of the target compounds.

| **Group** | **Compound** | **Molecular weight (g mol^-1^)** | **pK_a_** | **Log K_ow_** | **Structure** |
| --- | --- | --- | --- | --- | --- |
| Macrolides | **Roxithromycin (RXM)** | 837.0 | 9.08^a^, 12.45^a^ | 2.75^f^ | 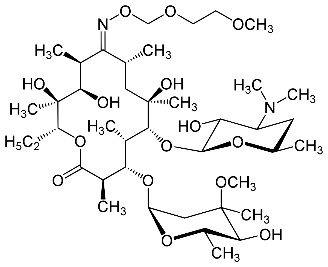 |
|  | **Azithromycin (AZM)** | 749.0 | 9.57^a^, 12.43^a^ | 4.02^f^ | 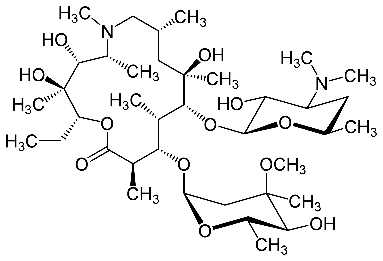 |
|  | **Erythromycin (ERY)** | 733.9 | 8.38^a^, 12.44^a^ | 3.06^f^ | 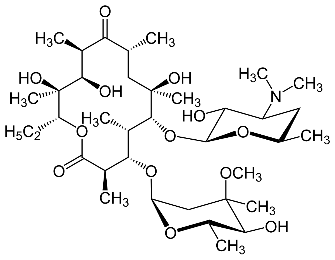 |
|  | **Clarithromycin (CLM)** | 747.9 | 8.38^a^, 12.46^a^ | 3.16^f^ | 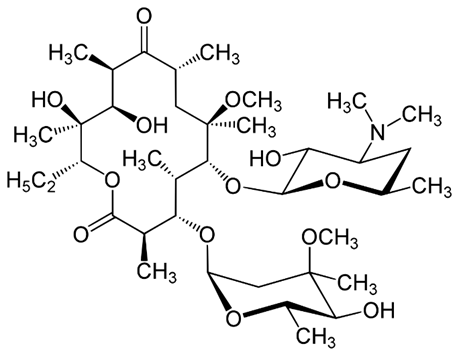 |
|  | N-desmethylclarithromycin (DM-CLM) | 733.9 | 13.08^b^ | - | 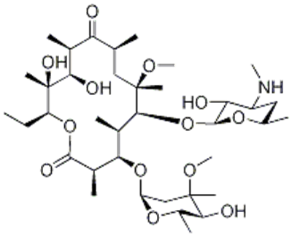 |
| Fluoroquinolones | **Norfloxacin (NOR)** | 319.3 | 5.77ª, 8.68^a^ | −0.875^c^ | 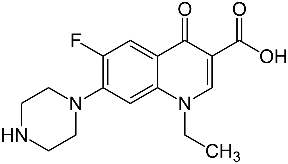 |
|  | **Enrofloxacin (ENR)** | 359.4 | 5.69^a^, 6.68^a^ | 0.648^c^ | 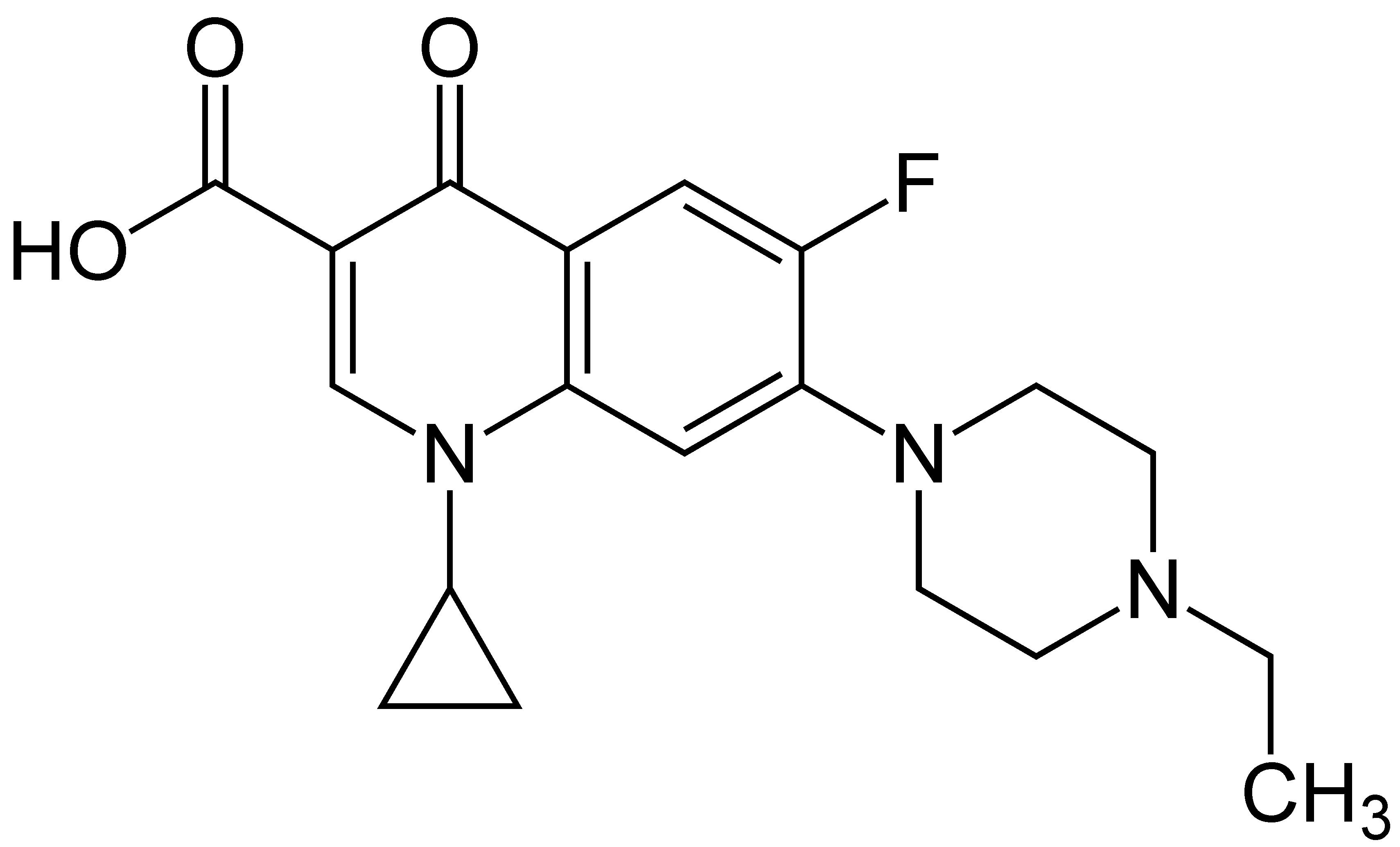 |
|  | **Ciprofloxacin (CIP)** | 331.3 | 5.76^a^, 8.68^a^ | 1.32^d^ | 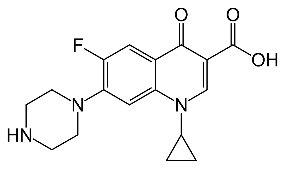 |
| Tetracyclines | **Tetracycline (TC)** | 444.4 | -2.2^a^, 8.24^a^ | -1.37^d^ | 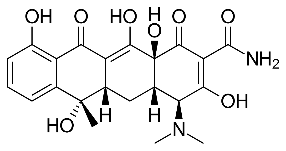 |
|  | 4-epitetracycline (EP-TC) | 444.4 | 4.8^b^, 8.0^b^ | -1.33^f^ | 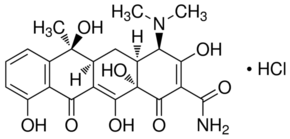 |
| Antifolates | **Trimethoprim (TMP)** | 290.3 | 7.16^a^, 17.33^a^ | 0.91^g^ | 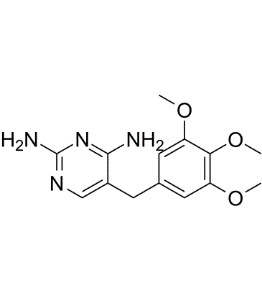 |
|  | 4-hydroxytrimetoprim (4-OH-TMP) | 306.3 | 8.18^b^ | - | 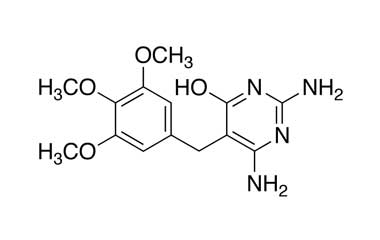 |
|  | 3-desmethyltrimethoprim (DM-TMP) | 276.3 | 9.40^b^ | - | 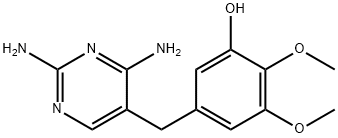 |
| Sulfonamides | **Sulfamethoxazole (SMX)** | 253.3 | 1.97^a^, 6.16^a^ | 0.89^g^ | 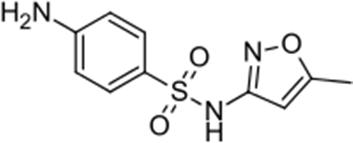 |
|  | N^4^-acetylsulfamethoxazole (AcSMX) | 295.3 | 5.54^e^ | 1.18^e^ | 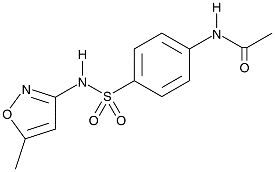 |
|  | Sulfamethoxazole N^4^-glucoside (SMX-GL) | 415.4 | - | - | 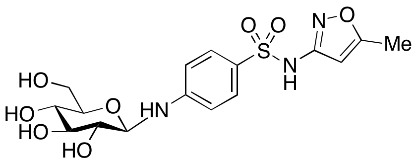 |
|  | **Sulfadiazine (SDZ)** | 250.3 | 2.01^a^, 6.99^a^ | −0.09^g^ | 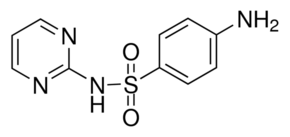 |
|  | N^4^-acetylsulfadiazine (AcSDZ) | 292.3 | 6.1^h^ | 0.39^f^ | 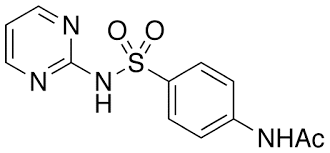 |
|  | **Sulfamethazine (SMZ)** | 278.3 | 2.04^a^, 6.99^a^ | 0.25^g^ | 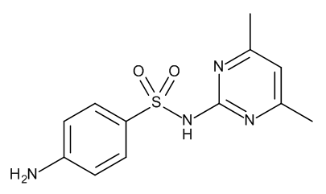 |
|  | N^4^-acetylsulfamethazine (AcSMZ) | 320.4 | 7.16^i^ | 1.48^f^ | 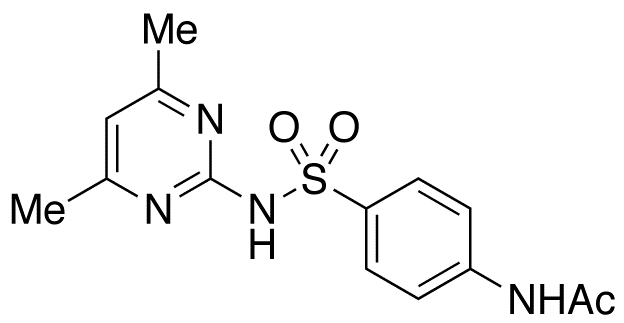 |

Parent compounds are marked in bold; Abbreviations are written in brackets; ^a^: https://go.drugbank.com/; ^b^: https://www.chemicalbook.com/; ^c^: Chen et al., 2020; ^d^: Li et al., 2018a; ^e^: Malvar et al., 2019; ^f^: Li et al., 2018b; ^g^: Lee et al., 2021; ^h^: Scudi and Plekss, 1958; ^i^: Yuan et al., 2019.

**Table S2.** Box–Behnken design matrix for the optimisation of clean-up sorbent type and amount.

| **Experiment** | **C18 amount (g)** | **PSA amount (g)** | **Florisil® amount (g)** |
| --- | --- | --- | --- |
| 1 | 0.8 | 0.4 | 0.8 |
| 2 | 0.8 | - | 0.4 |
| 3 | 0.4 | 0.4 | 0.4 |
| 4 | 0.4 | 0.8 | - |
| 5 | - | 0.8 | 0.4 |
| 6 | - | 0.4 | - |
| 7 | 0.8 | 0.8 | 0.4 |
| 8 | 0.4 | - | - |
| 9 | - | - | 0.4 |
| 10 | 0.4 | 0.4 | 0.4 |
| 11 | 0.4 | - | 0.8 |
| 12 | 0.4 | 0.8 | 0.8 |
| 13 | 0.4 | 0.4 | 0.4 |
| 14 | - | 0.4 | 0.8 |
| 15 | 0.8 | 0.4 | - |

**Table S3.** Box–Behnken design matrix for the optimisation of extraction solvent volume, time of extraction and number of extraction cycles.

| **Experiment** | **Solvent volume (mL)** | **Time of extraction (min)** | **Number of extractions cycles** |
| --- | --- | --- | --- |
| 1 | 3 | 15 | 2 |
| 2 | 3 | 5 | 2 |
| 3 | 7 | 10 | 1 |
| 4 | 3 | 10 | 3 |
| 5 | 5 | 15 | 1 |
| 6 | 5 | 5 | 1 |
| 7 | 7 | 15 | 2 |
| 8 | 7 | 5 | 2 |
| 9 | 5 | 15 | 3 |
| 10 | 5 | 10 | 2 |
| 11 | 5 | 5 | 3 |
| 12 | 7 | 10 | 3 |
| 13 | 5 | 10 | 2 |
| 14 | 3 | 10 | 1 |
| 15 | 5 | 10 | 2 |

**Table S4.** Method application to bird faeces samples from Doñana National Space.

| Compound | *Ciconia ciconia* (ng g^-1^ dw) | | | | | | | | | | | | | | |
| --- | --- | --- | --- | --- | --- | --- | --- | --- | --- | --- | --- | --- | --- | --- | --- |
|  | S1 | S2 | S3 | S4 | S5 | S6 | S7 | S8 | S9 | S10 | S11 | S12 | S13 | S14 | S15 |
| RXM | 0.49 | 0.57 | 0.55 | 0.59 | 1.15 | 1.91 | 0.42 | 0.61 | 0.28 | 0.95 | 0.37 | 0.21 | 0.73 | 0.34 | 0.40 |
| AZM | 0.80 | 0.83 | 0.77 | 0.78 | 0.71 | 0.68 | 0.95 | 1.08 | 1.02 | 1.52 | 2.44 | 0.92 | 1.04 | 0.66 | 0.68 |
| ERY | - | - | - | - | - | - | - | - | - | - | - | - | - | - | - |
| CLM | <MQL | <MQL | <MQL | <MQL | <MQL | <MQL | 0.42 | <MQL | <MQL | <MQL | <MQL | <MQL | 0.13 | 0.06 | 0.05 |
| DM-CLM | - | - | - | - | - | - | 0.34 | - | - | - | - | - | 0.08 | - | - |
| NOR | 17.52 | 15.91 | 13.37 | 9.24 | 7.99 | 7.43 | 21.59 | 14.96 | 199.27 | 19.44 | 15.47 | 17.45 | 21.70 | 10.73 | 10.87 |
| ENR | 3.85 | 4.96 | 2.93 | 3.03 | 3.56 | 5.44 | 2.93 | 4.54 | 5.97 | 19.22 | 22.88 | - | 5.69 | 2.92 | 2.93 |
| CIP | 28.27 | 24.44 | 19.81 | 18.02 | 14.32 | 40.84 | 28.22 | 47.04 | 26.29 | 24.54 | 43.22 | - | 22.10 | 29.64 | 31.25 |
| TC | - | - | - | - | - | - | - | - | - | - | - | - | - | - | - |
| EP-TC | - | - | 56.02 | 58.74 | 59.16 | - | - | - | - | - | - | 57.67 | - | - | - |
| TMP | 0.97 | 0.96 | 0.87 | 0.86 | 0.92 | 0.70 | 0.90 | 1.03 | 0.86 | 1.79 | 1.50 | 0.37 | 1.82 | 0.94 | 0.95 |
| 4-OH-TMP | - | - | - | - | - | - | - | - | - | - | - | - | - | - | - |
| DM-TMP | - | - | - | - | - | - | - | - | - | - | - | - | - | <MQL | <MQL |
| SMX | 2.14 | 2.36 | 300.62 | 25.18 | 2.81 | 73.55 | 16.43 | - | - | - | - | - | <MQL | <MQL | 12.57 |
| AcSMX | - | - | - | - | - | - | - | - | - | - | - | - | - | 2.54 | 2.57 |
| SMX-GL | - | - | - | - | - | - | - | - | - | - | - | - | - | - | - |
| SDZ | - | - | - | - | - | - | - | - | - | - | - | - | 6.53 | - | - |
| AcSDZ | - | - | - | - | - | - | - | - | - | - | - | - | - | - | - |
| SMZ | - | - | - | - | - | - | - | - | - | - | - | - | - | - | - |
| AcSMZ | - | - | - | - | - | - | - | - | - | - | - | - | - | - | - |

-: not detected; <MQL: lower than the limit of quantification of the method; Parent compounds are marked in bold.

**Table S4. Continued.**

| Compound | *Larus fuscus* (ng g^-1^ dw) | | | | | | | | *Chroicocephalus ridibundus* (ng g^-1^ dw) | | | |
| --- | --- | --- | --- | --- | --- | --- | --- | --- | --- | --- | --- | --- |
|  | S1 | S2 | S3 | S4 | S5 | S6 | S7 | S8 | S1 | S2 | S3 | S4 |
| RXM | 0.37 | 0.50 | 0.54 | 0.58 | 0.74 | 0.68 | 0.80 | 0.41 | 0.36 | 0.22 | 0.20 | 0.24 |
| AZM | 0.73 | 0.69 | 0.71 | 0.72 | 0.75 | 0.71 | 0.87 | 1.51 | - | - | - | - |
| ERY | - | - | - | - | - | - | - | - | - | - | - | - |
| CLM | <MQL | <MQL | <MQL | <MQL | 0.074 | 0.083 | 0.07 | 0.06 | <MQL | <MQL | <MQL | <MQL |
| DM-CLM | - | - | - | - | - | - | - | 0.09 | - | - | - | - |
| NOR | 9.24 | 8.21 | 10.54 | 10.73 | 8.63 | 12.10 | 11.72 | 17.52 | 3.87 | 4.27 | 4.44 | 7.43 |
| ENR | 2.51 | 2.92 | 5.18 | 2.79 | 4.54 | 2.96 | 2.88 | 2.79 | 7.37 | 27.24 | 11.08 | 6.31 |
| CIP | 9.15 | 8.24 | 10.51 | 10.74 | 8.63 | 12.06 | 15.35 | 15.74 | - | - | - | - |
| TC | - | - | - | - | - | - | - | - | - | - | - | - |
| EP-TC | - | - | - | - | - | - | - | - | - | - | - | - |
| TMP | 0.70 | 1.42 | 3.72 | 1.55 | 1.35 | 0.89 | 1.35 | 0.99 | 0.19 | 0.29 | 0.30 | 0.40 |
| 4-OH-TMP | - | - | - | - | - | - | - | - | - | - | - | - |
| DM-TMP | - | - | - | - | - | <MQL | - | - | - | - | - | - |
| SMX | - | 6.98 | 2.66 | 3.84 | 2.52 | 2.19 | 14.27 | 10.35 | - | - | - | - |
| AcSMX | - | - | - | - | - | - | 26.98 | 148.02 | - | - | - | - |
| SMX-GL | - | - | - | - | - | - | - | - | - | - | - | - |
| SDZ | - | - | - | - | - | - | 4.81 | - | <MQL | 4.26 | - | - |
| AcSDZ | - | - | - | - | - | - | - | - | - | - | - | - |
| SMZ | - | - | - | - | - | - | - | - | - | - | - | - |
| AcSMZ | - | - | - | - | - | - | - | - | - | - | - | - |

-: not detected; <MQL: lower than the limit of quantification of the method; Parent compounds are marked in bold.

**Figure S1.** MRM chromatogram of a matrix-matched calibration standard at 75 ng g^-1^ dw for each compound

**Supplementary material references**

Chemical BooK. Sourcing and Integrating Center of Chemicals Materials in China. Available on: https://www.chemicalbook.com/

Chen, S., Zhang, W., Li, J., Yuan, M., Zhang, J., Xu, F., Xu, H., Zheng, X., Wang, L., 2020. Ecotoxicological effects of sulfonamides and fluoroquinolones and their removal by a green alga (*Chlorella vulgaris*) and a cyanobacterium (*Chrysosporum ovalisporum*) Environ. Pollut. 263: 114554. <https://doi.org/10.1016/j.envpol.2020.114554>

DrugBank Online. Database for Drug and Drug Target Info. Available on: <https://go.drugbank.com/>

Lee, H.J., Kim, D.W., Chung, E.G., 2021. Strong links between load and manure and a comprehensive risk assessment of veterinary antibiotics with low K_OW_ in intensive livestock farming watersheds. Chemosphere. 279: 130902. https://doi.org/10.1016/j.chemosphere.2021.130902

Li, J., Zhang, K., Zhang, H., 2018a. Adsorption of antibiotics on microplastics. Environ. Pollut. 237: 460-467. <https://doi.org/10.1016/j.envpol.2018.02.050>

Li, S., Shi, W., Liu, W., Li, H., Zhang, W., Hu, J., Ke, Y., Sun, W., Ni, J., 2018b. A duodecennial national synthesis of antibiotics in China's major rivers and seas (2005–2016). Sci. Total Environ. 615: 906-917. <https://doi.org/10.1016/j.scitotenv.2017.09.328>

Malvar, J.L., Santos, J.L., Martín, J., Aparicio, I., Alonso, E., 2019. Routine analytical method for monitoring the main metabolites for a recurrent group of parabens and pharmaceuticals in wastewater and tap water. Anal. Bioanal. Chem. 411: 6625-6635. https://doi.org/10.1007/s00216-019-02035-2

Scudi, J.V., Plekss O.J., 1958. Chemotherapeutic Activity of Some Sulfapyridine-1-Oxides. Proc. Soc. Exp. Biol. Med. 97:3. <https://doi.org/10.3181/00379727-97-23830>

Yuan, S., Liu, Z., Yin, H., Dang, Z., Wu, P., Zhu, N., Lin, Z. Trace determination of sulfonamide antibiotics and their acetylated metabolites via SPE-LC-MS/MS in wastewater and insights from their occurrence in a municipal wastewater treatment plant. Sci. Total Environ. 653: 815-821. https://doi.org/10.1016/j.scitotenv.2018.10.417
